# Identification and Comprehensive Analysis of circRNA-miRNA-mRNA Regulatory Networks in A2780 Cells Treated with Resveratrol

**DOI:** 10.3390/genes15070965

**Published:** 2024-07-22

**Authors:** Weihua Zhu, Yuanting Zhang, Qianqian Zhou, Cheng Zhen, Herong Huang, Xiaoying Liu

**Affiliations:** 1 Department of Basic Medical Sciences, Clinical College of Anhui Medical University, Hefei 230031, China; 2 School of Life Sciences, Anhui Medical University, Hefei 230032, China

**Keywords:** resveratrol, cell proliferation, cell apoptosis, ovarian cancer

## Abstract

Ovarian cancer (OC) is one of the most commonplace gynecological malignancies. This study explored the effects of resveratrol (RES) on OC cell proliferation and apoptosis. Proliferation activity was measured for A2780 cells treated with RES for 24 h and 48 h at concentrations of 0, 10, 25, 50, 75, 100, 150, 200, and 300 µM. RNA sequencing (RNA-seq) was performed to analyze the circular RNA (circRNA), microRNA (miRNA), and messenger RNA (mRNA) expression spectrum. The differentially expressed genes included 460 circRNAs, 1988 miRNAs, and 1671 mRNAs, and they were subjected to analyses including Gene Ontology, the Kyoto Encyclopedia of Genes and Genomes (KEGG), and Reactome enrichment. We selected signaling pathways enriched in the cell processes by mRNA KEGG, comprehensively analyzed the circRNA-miRNA-mRNA regulatory network, and verified several miRNAs expressed in the regulatory network diagram using the quantitative real-time polymerase chain reaction. The data showed that the cell proliferation of A2780 cells treated with RES for 24 h or 48 h decreased with increasing concentrations of RES. The circRNA-miRNA-mRNA regulatory network that we constructed provides new insights into the ability of RES to inhibit cell proliferation and promote apoptosis in A2780 cells.

## 1. Introduction

Ovarian cancer (OC) is one of the most common malignant gynecological cancers and a significant cause of cancer-related deaths in women worldwide [1,2]. Because of its lack of early clinical symptoms, most patients develop late-stage OC before diagnosis [3]. Tumors scored as the same histological subtype may comprise different subtypes with distinct biological and molecular characteristics, providing unpredictability in the availability and application of treatment. Consequently, OC survival rates have not changed significantly for decades [4]. At present, surgery is the main treatment for OC; the patient prognosis is not optimistic, and the clinical recurrence rate is high [5]. Nevertheless, the molecular mechanism(s) of OC occurrence and dissemination are still to be fully elucidated. Moreover, the lack of disease-specific biomarkers and related therapeutic targets also represents major hurdles for improving treatment efficacy and the quality of life of patients with OC.

Although commonly used for treatment, advanced OC cases frequently exhibit resistance to platinum therapy. To overcome this challenge, traditional Chinese medicine has become increasingly popular in preventing and controlling OC, representing a safe alternative clinical treatment with various actions including inhibiting OC angiogenesis [6,7]. Traditional Chinese medicine can enhance the treatment efficacy for multi-drug resistance, especially for patients with chemotherapy-resistant OC [8]. Resveratrol (RES), a Chinese medicinal extract, has a wide range of antitumor properties on several types of cancer cells. The role of RES in controlling different human malignancies has been reported, including many common female cancers (breast, cervix, ovaries, and uterus) along with cancers of the bladder, blood, brain, esophagus, eye, gastrointestinal system, head and neck, kidney, liver, lung, oral cavity, prostate, skin, and thyroid [9,10,11]. It has been reported that RES induces OC cell apoptosis by inhibiting proliferation, invasion, and migration, regulating OC cell autophagy, interfering with related signaling pathways, and reversing multi-drug resistance [12,13,14].

Cell proliferation and apoptosis are important in tumor occurrence and development. Currently, research on the mechanism of RES treatment in OC is insufficient, and the mechanisms of cell proliferation and apoptosis in OC cells are still unclear. A microRNA (miRNA) is a single-stranded RNA molecule that is approximately 18–25 nucleotides in length. At the post-transcriptional level, miRNAs can interact with complementary sequences within the 3′-untranslated region of specific messenger RNAs (mRNAs), thus selectively targeting mRNA expression. For example, overexpressed miR-744-5p activates the intrinsic apoptotic pathway in the OVCAR3 and SKOV3 OC cell lines, along with both non-resistant and cisplatin-resistant A2780 cells, leading to cell death [15]. In addition, miR-612 suppresses OC progression by directly targeting NIN1/RPN12 binding protein 1 homolog (*NOB1*) mRNA, but it is downregulated in OC tissues from patients as well as different OC cell lines [16]. Analyses of the immune microenvironment have shown that hsa-miR-675-3p is crucial in OC [1], while miR-29c-3p overexpression in OC cells also inhibits their proliferation and migration [17]. Following progress in cellular and molecular biology mechanisms, the role of post-transcriptional modifications of non-coding RNAs (ncRNAs) in mRNA regulation has received increasing attention. Circular RNAs (circRNAs) are endogenous ncRNAs implicated in cancers and have many functions in the development and progression of cancer through different mechanisms [18,19,20]. Evidence suggests that interactions between miRNAs and circRNAs are broadly important in human disease pathogenesis. Akin to miRNA-mRNA interactions, circRNAs interact with miRNAs to alter their actions against target genes, effectively enhancing the expression of miRNA targets. The regulatory networks formed by circRNAs, miRNAs, and mRNA play crucial roles in cancer progression [21]. For example, circRNA-MYLK promotes a malignant phenotype in OC cells through regulating miRNA-652 levels, and notably, circRNA-MYLK expression correlates with worse pathological staging and outcomes in patients with OC [22]. Circ-LOPD2, which binds and sequesters miR-378, promotes the proliferation of OC cells, possibly promoting the progression of OC [23]. Additionally, hsa_circ_0013958 knockdown suppresses the proliferation, migration, and invasion of OC cells with elevations in cell apoptosis rates [24]. However, circRNAs do not always function as oncogenes, as occurs with circ-ITCH, which shows diminished levels in OC and otherwise suppresses OC cell proliferation and promotes apoptosis through sponging miR-10a [25].

Despite numerous preclinical studies investigating the therapeutic potential of RES, this research has seldom been translated into clinical practice with limited applications to anti-cancer clinical trials [26]. Basic experiments using RES to treat OC are relatively common, but its molecular mechanism needs to be explored. The way in which circRNA-miRNA-mRNA interactions occur in A2780 OC cells treated with RES is unknown. Therefore, this study mainly focused on identifying specific circRNAs, miRNAs, and mRNAs influenced by RES treatment in A2780 cells. The circRNA, miRNA, and mRNA expression profiles of A2780 cells treated with and without RES were analyzed. Gene Ontology (GO) enrichment, Kyoto Encyclopedia of Genes and Genomes (KEGG) pathway analyses, Disease Ontology enrichment, and Reactome enrichment were implemented to uncover the underlying biological processes affected by RES treatment. Several potential competing endogenous RNA (ceRNA) modules involving circRNA-miRNA-mRNA interactions were identified to reveal the overall regulatory network. These data further reveal the mechanism of cell proliferation and apoptosis in A2780 cells, providing a resource for future applications of RES in clinical medicine.

## 2. Materials and Methods

### 2.1. Cell Culture

Human A2780 OC cells (iCell Bioscience Inc., Shanghai, China) were cultured in RPMI-1640 medium (Gibco; Grand Island, NY, USA) containing 10% fetal bovine serum (Gibco; Grand Island, NY, USA) and 1% antibiotic–antimycotic solution (Gibco; Grand Island, NY, USA). Cells were maintained at a 37 °C temperature in a humidified atmosphere supplemented with 5% CO_2_. When cell confluence reached 80%, culture media were removed and replaced with fresh media containing the indicated concentrations of RES (0, 10, 25, 50, 75, 100, 150, 200, and 300 µM) (Solarbio; Beijing, China). Cells were cultured for 24 h or 48 h with triplicate wells used for different RES concentrations and times.

### 2.2. Cell Proliferation (Cell Counting Kit-8 Assay)

Cell proliferation assays were performed using the Cell Counting Kit-8 (CCK-8; biosharp; Hefei, China) according to the manufacturer’s instructions. Briefly, A2780 cells were seeded into 96-well plates and treated with RES (0, 10, 20, 50, 75, 100, 150, 200, or 300 µM) for 24 h or 48 h. CCK-8 solution was then added to each well and further incubated before measuring optical density (OD) values using a microplate reader (DeTie; Nanjing, China) at 450 nm. Presented assays were conducted at least three times with similar results.

### 2.3. RNA Sample Detection, Library Construction, and Sequencing

Human A2780 cells subjected to control (0 µM RES) and test conditions (75 µM RES) were used for RNA-seq analysis. After extracting total RNA using the TRIzol reagent kit (Invitrogen; Carlsbad, CA, USA) according to the manufacturer’s protocol, the purity and concentration of the extracted RNA were evaluated by the NanoDrop 2000 optical test. RNA quality checks were performed on an Agilent 2100 Bioanalyzer (Agilent Technologies; Palo Alto, CA, USA) or by electrophoresis on RNase-free agarose gels. Only qualified samples were used for sequencing from three biological replicates for each treatment group.

Full transcription group sequencing technology was used to build two sequencing libraries, the small RNA (sRNA) library, and a chain-specific library, with the NucleoScan RNA removed. The miRNA sequence information was obtained from the sRNA library. The sRNA Sample Pre Kit was used to build the library; T4 RNA ligase 2 (truncated) was used to ligate the sRNA 3′ and 5′ ends. rRNA was removed from the chain-specific library to obtain mRNA and circRNA sequence information. An Epicentre Ribo-Zero kit was used to remove ribosomal RNA (rRNA). Complementary DNA (cDNA) was synthesized by adding fragmentation buffer to randomly break the rRNA-depleted RNAs, which were then used as templates for first strand synthesis of cDNA using random hexamer primers; the second chain of cDNA was then synthesized. Next, after purifying the cDNA fragments with VAHTS DNA Clean Beads (Vazyme; Nanjing, China), products were end-repaired, added to poly(A) sequences, and ligated with Illumina sequencing adapters. Second-strand cDNA was digested with uracil-N-glycosylase before purification with VAHTS DNA Clean Beads, amplification was performed by PCR, and sequencing was performed using an Illumina HiSeq 2500 instrument (Gene Denovo Biotechnology Co.; Guangzhou, China).

### 2.4. Identification and Quantification of CircRNAs

High-quality sequences were obtained from the DNA sequencing data through filtering via fastp (version 0.18.0) [27]. The resulting short sequence reads were then mapped to the rRNA database using the Bowtie2 alignment tool (version 2.2.8) [28]. The rRNA-removed reads were mapped to the reference genome using HISAT2 (version 2.1.1) [29] to remove rRNA-mapped sequences, with the remaining sequences being used for alignment and analysis. Further data filtering involved alignments against the reference genome, removing mapped reads while collecting unmapped reads for putative circRNA identification. From the unmapped reads, we then extracted and aligned 20 mers sequences from both ends against the reference genome to identify unique anchor positions within the splice site. Anchor reads aligned in reverse (head-to-tail) orientations were used to define circRNA splicing events; these sequences were subjected to circRNA identification [30]. The anchor alignments were extended to define the complete reads and identify breakpoints flanked by GU/AG splice sites. For data inclusion, each candidate circRNA required the detection of two or more unique back-spliced reads from one or more samples. CircRNA expression levels are described as reads per million mapped reads. Differentially expressed circRNAs were identified using the edgeR package (v3.20.9) using threshold values of |log_2_ (fold-change)| > 1 and q < 0.05.

### 2.5. Identification and Quantification of mRNA Expression

Reads of low quality containing adapter sequences and/or undetermined bases were filtered out from the raw data using fastp (version 0.18.0) [27], with high-quality (clean) reads being obtained following the removal of rRNA sequences. After building an index of the reference genome, paired-end clean reads were mapped to the reference using HISAT2 (version 2.1.0) [29] with “-RNA-strandness RF” and default settings for the other parameters. Transcript reconstruction was performed using StringTie software (version 1.3.4) [31] combined with HISAT2, allowing for the identification of new genes. The mapped reads of each sample were assembled in StringTie v1.3.1 using a reference approach. For each transcribed region, the value of the fragment per kilobase of transcript per million mapped reads was calculated to quantitate both abundance and variation using RSEM software (version 1.2.19) [32]. RNA differential expression analysis was performed using the DESeq2 software (version 1.20.0) [33] between different groups (and edgeR [34] between two samples). Differentially expressed mRNAs were identified using the criteria of |log_2_ (fold-change)| > 1 and q < 0.05.

### 2.6. Analysis of miRNA Expression

Raw reads obtained from the miRNA library were filtered using Cutadapt 1.8.1. All clean tags were aligned with small RNA genes in the GenBank database (release 209.0) to identify and remove rRNA, small conditional RNA, small nucleolar RNA, small nuclear RNA, and transfer RNA. All clean tags were searched against the miRBase database (release 22) to identify miRNAs defined in other species through prior studies. Since miRNA sequences of certain species are not included in the miRBase database, the alignment of candidate miRNAs with those in other species can be a useful identification strategy. All unannotated tags were aligned to the reference genome, and mirdeep2 software (version 2.0.0.7) was used to identify novel miRNA candidates based on genome position and predicted hairpin structures. Calculated miRNA expression levels were normalized as transcripts per million, with differential expression analysis of miRNAs being performed using edgeR package (version 3.20.9). We defined differentially expressed miRNAs as those with a ≥ 2-fold change and *p* < 0.05.

### 2.7. Bioinformatic Analyses

GO enrichment and KEGG pathway analyses were performed to analyze the advanced functions of differentially expressed genes, miRNA-targeted genes, and circRNA parental genes. GO analysis uses the GO resource (http://www.geneontology.org, accessed on 1 January 2023), which labels genes with functions, including molecular functions, biological processes, and cellular components. The KEGG database (version Release 101) outputs annotations related to signal transduction and disease pathways, providing gene-level information for further functional analysis. *p* < 0.05 was considered statistically significant enrichment.

In the constructed circRNA-miRNA-mRNA networks, we selected mRNAs enriched in cell processes based on a KEGG analysis. We used Cytoscape software (version 3.9.1) to construct a circRNA-miRNA-mRNA network targeting these mRNAs and circRNAs and miRNAs differentially expressed in RNA-seq.

### 2.8. Real-Time Polymerase Chain Reaction

A LightCycler96 instrument (Roche; Basel, Switzerland) was used to confirm expression level changes in differentially expressed miRNAs. cDNA was synthesized using EasyScript One-Step genomic DNA Removal and cDNA Synthesis SuperMix (Transgen; Beijing, China). The primer sequences used to detect specific miRNAs are listed in Table 1. Quantitative real-time polymerase chain reactions (RT-qPCR) were performed in 20 µL reaction mixtures containing 10 µL of 2× Top Green EX-Taq Mix (Transgen; Beijing, China), 2 µL of cDNA, 7 µL of ddH_2_O, and 0.5 µL of forward and reverse primers. U6 transcript levels were used as housekeeping references to normalize miRNA expression levels, with normalized expression values being calculated via the 2^−ΔΔCt^ method. Each sample analysis was performed in triplicate to reduce experimental errors.

### 2.9. Statistical Data Analysis

RT-qPCR data represent mean ± standard deviation of triplicate determinations. GraphPad Prism software version 5.0 (San Diego, CA, USA) was used to analyze statistical differences between groups using Student’s two-tailed *t*-test. *p*-values < 0.05 were considered statistically significant, marked as * *p* < 0.05 and ** *p* < 0.01.

## 3. Results

### 3.1. Proliferation of A2780 Cells Treated with Resveratrol

**Figure 1:**
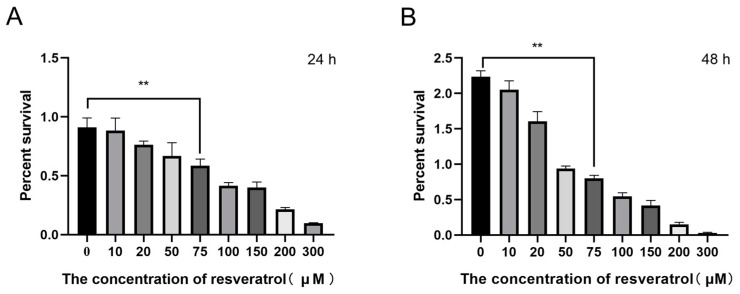
RES inhibits the proliferation of A2780 cells. (**A**) A2780 cells treated with 0, 10, 25, 50, 75, 100, 150, 200, and 300 µM of RES for 24 h, with cell proliferation detected using the Cell Counting Kit 8 (CCK-8). (**B**) A2780 cells treated with RES for 48 h, with cell proliferation detected by CCK-8. ** *p* < 0.01.

Cell proliferation assays were first applied to determine the effects of different concentrations of RES (0, 10, 25, 50, 75, 100, 150, 200, and 300 µM) on A2780 cells over 24 h (Figure 1A) and 48 h of treatment (Figure 1B). The highest rate of A2780 cell proliferation occurred in the 0 µM group (Figure 1A,B); the lowest rate of cell proliferation was observed in the 300 µM group (Figure 1A,B). Whether cultured for 24 h or 48 h, the rate of A2780 cell proliferation decreased with the increasing RES concentration. The rate of A2780 cell proliferation in the 75 µM group was approximately 50% at 24 h. Therefore, we selected two groups of A2780 cells for RNA-seq: a control group (0 µM RES) and an experimental group (treated with 75 µM of RES for 24 h).

### 3.2. miRNA Sequencing (miRNA-seq) and Data Analysis

**Figure 2:**
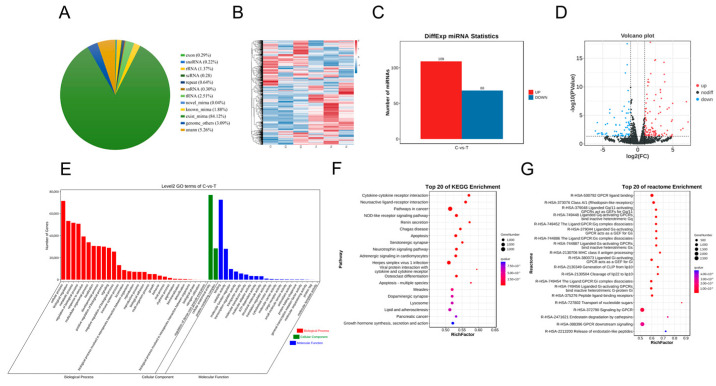
Heatmap and function enrichment map of differentially expressed miRNAs in A2780 cells treated with resveratrol. (**A**) Classification pie chart of all small RNAs. (**B**) Heatmap of all expressed miRNAs in A2780 treated with resveratrol. (**C**) Differentially expressed miRNA statistics. (**D**) Volcano plots of differentially expressed miRNAs. (**E**) Gene Ontology analysis of differentially expressed miRNAs. (**F**) KEGG analysis of differentially expressed miRNAs. (**G**) Reactome analysis of differentially expressed miRNAs.

A total of 1988 miRNAs (Supplementary Table S1) were identified between the control and 75 µM RES groups; known and exist miRNAs accounted for most sRNAs (approximately 86.00%); and rRNA accounted for 1.37% (Figure 2A). A total of 1069 existing miRNAs, 524 known miRNAs, and 395 novel miRNAs were included, as shown in the heat maps in Figure 2B. A total of 177 differentially expressed miRNAs were screened using edgeR package (version 3.20.9), including 109 upregulated and 68 downregulated miRNAs (Figure 2C; Supplementary Table S2). Differentially expressed miRNAs are shown as cluster volcanoes (Figure 2D). The miRanda (Version 3.3a) and TargetScan software (Version 7.0) packages were used to predict the miRNA target genes; 87298 target genes were differentially expressed. The functional annotation of the differentially expressed miRNA target genes showed that the GO biological process was enriched mainly in transcription, cellular processes, and biological regulation. Cellular components were enriched in anatomical entities and protein-containing complexes. Molecular functions were enriched mainly in binding and catalytic activity (Figure 2E). The KEGG pathways were enriched mainly in cancer, herpes simplex virus 1 infection, and related pathways (Figure 2F). The Reactome enrichment pathway identified genes mainly for signaling by G protein-coupled receptors (GPCR) and GPCR downstream signaling (Figure 2G).

### 3.3. CircRNA Sequencing (circRNA-seq) Analysis

**Figure 3:**
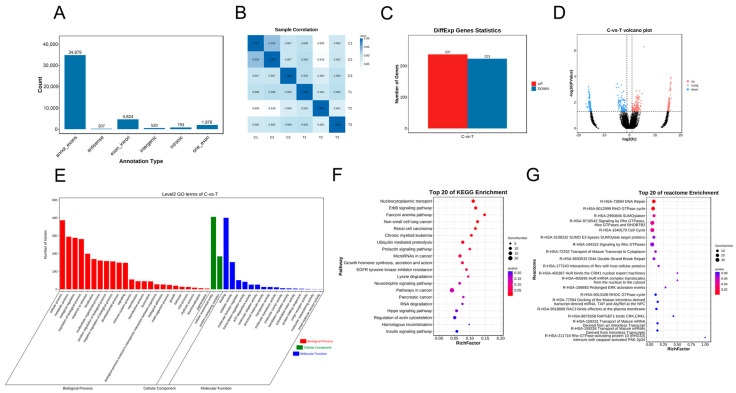
Heatmap and function enrichment map of differentially expressed circRNAs in A2780 cells treated with resveratrol. (**A**) Classification pie chart of all circRNAs. (**B**) Sample correlation analysis of all circRNAs in A2780 treated with resveratrol. (**C**) Differentially expressed circRNA statistics. (**D**) Volcano plots of differentially expressed circRNAs. (**E**) Gene Ontology analysis of differentially expressed circRNAs. (**F**) KEGG analysis of differentially expressed circRNAs. (**G**) Reactome analysis of differentially expressed circRNAs.

A total of 42,999 circRNAs (Supplementary Table S3) were identified in the two groups (Figure 3A). Importantly, comparisons of the three replicates for each group showed strong correlations (Figure 3B). A total of 460 differentially expressed circRNAs were recorded, including 237 upregulated and 223 downregulated circRNAs (Figure 3C; Supplementary Table S4); the cluster volcanoes of the differentially expressed circRNAs are shown in Figure 3D. The GO analysis showed that the enriched biological processes included cellular and metabolic processes. The cellular components were enriched in cellular anatomical processes and protein-containing complexes; enriched molecular function entries included binding and catalytic activity (Figure 3E). The KEGG pathways were enriched across related signaling pathways, including pathways in cancer and nucleocytoplasmic transport (Figure 3F). The Reactome enrichment pathway identified entries used mainly for signaling by Rho GTPases, Miro GTPases, and RHOBTB3 and in the cell cycle (Figure 3G).

### 3.4. mRNA Sequencing (mRNA-seq) and Analysis

**Figure 4:**
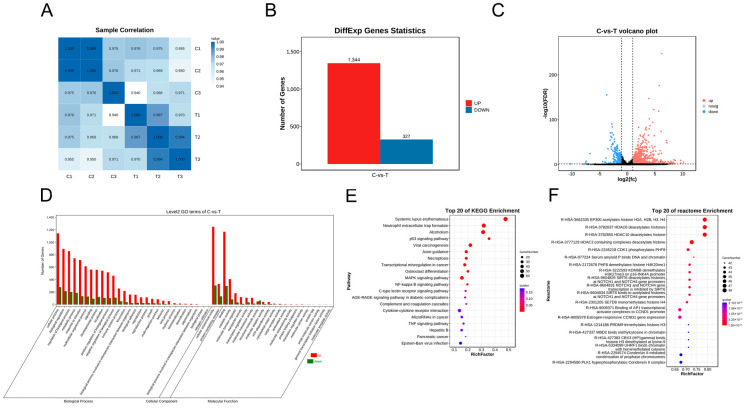
Heatmap and function enrichment map of differentially expressed mRNAs in A2780 cells treated with resveratrol. (**A**) Sample correlation analysis of all mRNAs in A2780 treated with resveratrol. (**B**) Differentially expressed mRNA statistics. (**C**) Volcano plots of differentially expressed miRNAs. (**D**) Gene Ontology analysis of differentially expressed mRNAs. (**E**) KEGG analysis of differentially expressed mRNAs. (**F**) Reactome analysis of differentially expressed mRNAs.

A total of 20,305 mRNAs (Supplementary Table S5) were identified. A sample correlation analysis showed that the three replicates of each group were highly correlated (Figure 4A). A total of 1671 differentially expressed mRNAs were recorded, including 1344 upregulated and 327 downregulated mRNAs (Figure 4B; Supplementary Table S6); cluster volcanoes of the differentially expressed mRNAs are shown in Figure 4C. A GO analysis revealed that the enriched biological processes included cellular processes, biological regulation, and the regulation of biological processes. Cellular component enrichment items included cellular anatomical entities and protein-containing complexes. The molecular function enrichment entries included binding and catalytic activity (Figure 4D). The KEGG pathways were enriched for systemic lupus erythematosus, neutrophil extracellular trap formation, and alcoholism (Figure 4E). The Reactome enrichment pathway identified items used mainly in EP300 to acetylate histones H2A, H2B, H3, H4, and HDAC8, which deacetylates histones (Figure 4F).

### 3.5. Construction of circRNA-miRNA-mRNA Regulatory Networks

**Figure 5:**
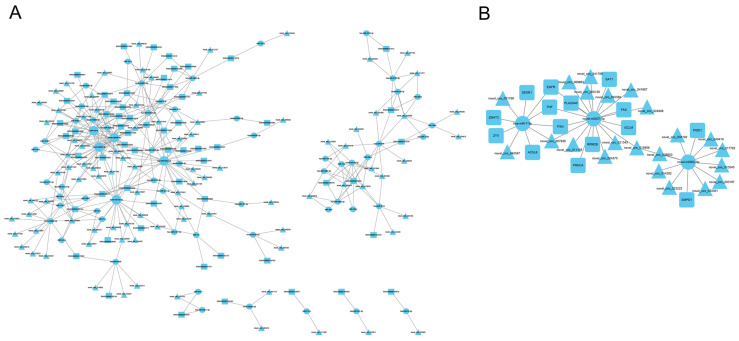
(**A**) Regulatory networks of miRNA, mRNA, and circRNA. Triangle nodes represent circRNA, circular nodes represent miRNA, and square nodes represent circRNA. (**B**) Regulatory network analysis identifies three miRNA-related ceRNA pairs.

We analyzed the results of the significant differences in KEGG mRNAs and identified four signaling pathways related to cell processes: the p53 signaling pathway, necroptosis, ferroptosis, and focal adhesion. For the mRNAs in these pathways and the corresponding circRNAs and miRNAs (Supplementary Table S7), we used Cytoscape to build circRNA-miRNA-mRNA regulatory networks (Figure 5A). We screened three miRNA-related ceRNA pairs to construct a circRNA-miRNA-mRNA regulatory network diagram involving novel m0007-5p, novel m0002-3p, and has-miR-7-5p (Figure 5B).

### 3.6. RT-qPCR Verification of Differentially Expressed miRNAs in A2780

**Figure 6:**
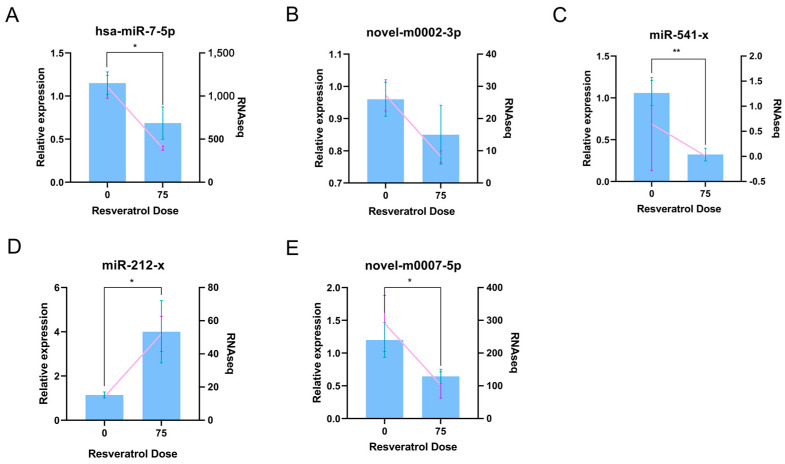
RT-qPCR detection of differentially expressed miRNAs. RT-qPCR and RNA-seq detection of hsa-miR-7-5 (**A**), novel-m0002-3p (**B**), miR-541-x (**C**), miR-212-x (**D**) and novel-m0007-5p (**E**). The blue column represents the qPCR results (* *p* < 0.05; ** *p* < 0.01); the pink lines represent the RNA-seq results.

To verify the integrity of the sRNA-seq findings, we independently performed RT-qPCR analyses on A2780 cells treated with RES to measure expression level changes in key miRNAs. We selected five miRNAs from the circRNA-miRNA-mRNA regulatory network (Figure 5A) to validate the sRNA-seq results. The five differentially expressed miRNAs are listed in Table 2. The relative expression levels of the validated miRNAs are shown in Figure 6. The RT-qPCR results show good consistency with the sRNA-seq findings.

## 4. Discussion

Natural compounds, such as RES, are used as auxiliary anticancer therapies to modulate diverse targets and signaling pathways [26,35]. RES can inhibit apoptosis and promote the proliferation and differentiation of pre-osteoblast MC3T3-E1 cells. The Traditional Chinese Medicine Systems Pharmacology Database and Analysis Platform were used to find the gene targets of RES; the potential target genes were enriched by GO, KEGG, and Reactome [36]. It has been reported that RES inhibits cell proliferation, promotes apoptosis, and regulates cell cycle progression and signaling pathways in HeLa and B cells [37,38,39,40]. RES significantly inhibited the proliferation, migration, and invasion of A2780 and SKOV3 OC cells, and it can exert antitumor effects in human and mouse OC cells by activating immunity and inducing the immunogenic death of cancer cells [41,42] while also inducing apoptosis, growth arrest, and autophagy in human OC cells by activating the adenosine monophosphate-activated kinase (AMPK)/mTOR signaling pathway and inhibiting glycolysis [43].

In our study, we analyzed the dose- and time-dependent effects of RES on A2780 OC cells. RES was found to significantly inhibit A2780 cell proliferation with the highest concentration of 300 µM RES used, producing near-complete growth inhibition. To gain further insights regarding the actions of RES on A2780 cell proliferative and apoptotic responses, we performed RNA sequencing on the control and 75 µM RES-treated groups, particularly to obtain information on the roles of circRNAs, miRNAs, and mRNAs.

CircRNA-miRNA-mRNA interaction networks are known to be dysregulated in the pathogenesis of many diseases, including diabetes, breast cancer, and endometrial cancer [44,45,46,47]. mRNAs, miRNAs, and circRNAs play crucial roles in the progression of breast cancer through a variety of biological effects and mechanisms; circRNAs indirectly regulate mRNA expression corresponding to the downstream target genes of miRNA through endogenous RNA competition, thereby promoting the progression of breast cancer [21]. A circRNA-miRNA-mRNA regulatory network was constructed in osteoarthritis, identifying three new differentially expressed circRNAs as novel osteoarthritis biomarkers [48]. mRNA, miRNAs, and circRNAs play important roles in OC progression [49,50].

Research on the network regulation of circRNA-miRNA-mRNA in OC cells is limited. In A2780 cells treated with RES, we observed 1344 upregulated mRNAs, 327 downregulated mRNAs, 237 upregulated circRNAs, 223 downregulated circRNAs, 109 upregulated miRNAs, and 68 downregulated miRNAs. We further explored the potential functions of differentially expressed circRNAs, miRNAs, and mRNAs in A2780 cells with RES treatment by GO and KEGG functional enrichment analyses. The pathways identified in this study included the p53 signaling pathway, necroptosis, ferroptosis, focal adhesion, and genes related to cancer pathways.

The p53 signaling pathway is associated with cell proliferation, apoptosis, and the cell cycle and it is regulated by a variety of factors [51,52,53,54]. Necroptosis and ferroptosis, also known as regulated necrotic cell death, have been extensively studied and shown to be critical for cancer treatment outcomes [55,56,57,58]. Cancer stem cells (CSCs) are the root cause of OC recurrence and drug resistance. The enrichment of the CSC gene pathway associated with drug resistance in OC suggests that focal adhesion signal transduction may play an important role in CSC gene-mediated drug resistance [59]. *SPTBN2* may regulate the proliferation, invasion, and migration of endometrial OC cells through ITGB4-mediated focal adhesion and extracellular matrix receptor signaling pathways [60]. Because of the importance of the circRNA-miRNA-mRNA interaction network in diseases, we used Cytoscape to build a circRNA-miRNA-mRNA regulatory network based on the mRNA in these pathways and the corresponding circRNAs and miRNAs. We found that the mechanism by which RES inhibits the proliferation of A2780 cells is complex.

RES induces the degradation of Gal-3 by upregulating miR-424-3p and mediating apoptosis in tumor cells [61]. MiRNAs fulfill key roles in maintaining cellular homeostasis, while their dysregulation in disease states also serves to drive various pathogenic phenotypes, for example, to mediate the metabolic reprogramming of tumors [62]. Indeed, numerous miRNAs have been shown to participate in tumorigenesis, both in oncogenic and tumor suppressor capacities. On this basis, selected miRNAs identified in our network analysis attracted our attention, including hsa-miR-7-5p, miR-212-x, novel-m0007-5p, novel-m0002-3p, and miR-541-X. For instance, hsa-miR-7-5p was shown to target *SPC24* expression in hepatocellular carcinoma cell lines, suppressing their proliferation and migration while also promoting apoptosis [63]; however, the regulatory actions of hsa-miR-7-5p in A2780 OC cells remains to be defined. MiR-212 and miR-541 have been studied in a variety of cancers, including OC, pancreatic cancer, nasopharyngeal cancer, breast cancer, gastric cancer, and bladder cancer. In certain cancers, miR-212 has greater expression in healthy people than in patients. Moreover, it can regulate cancer cell proliferation and apoptosis by targeting different genes, including mitogen-activated protein kinase kinase kinase 3 (*MAP3K3*), SRY-box transcription factor 4 (*SOX4*), and methyltransferase like 3 (*METTL3*). MiR-212 is regulated by nuclear factor IA (NFIA) and hypoxia-inducible factor-1α (HIF-1α) and has been identified as a potential biomarker for cancer diagnosis and treatment [64,65,66,67,68,69]. In addition to targeting molecules such as stromal interaction molecule 1 (*STIM1*), miR-541 is regulated by Circ_0085616, CircRTN4IP1, and CircMTO1 [70,71,72]. Hsa-miR-7-5p and novel-m0007-5p co-target *TNF*, *EGFR*, and *PXN* and are regulated by novel_circ_007936. Novel-m0007-5p and novel-m0002-3p are miRNAs regulated by novel_circ_015956. However, their functions and regulation require further investigation.

Lastly, we must acknowledge the limitations of our study. Foremost, our data are largely based on RNA-seq, with many of the findings implied from bioinformatic tools. As such, these tools do not provide evidence for the various RNA-protein interaction networks that function in parallel with RNA–RNA interactions. Moreover, although the RT-qPCR results demonstrate good consistency between the expression of miRNAs detected by RNA-seq data, further work is necessary for the confirmation of the large number of gene expression changes detected. The existence and involvement of individual circRNAs and miRNAs also requires additional empirical testing. This may include but is not limited to the verification of the corresponding circRNA-miRNA-mRNA regulatory networks revealed as well as establishing the broader relevance of these findings in the in vivo setting.

In summary, the high-throughput sequencing results suggest that differentially expressed circRNAs and miRNAs may play crucial roles in the regulation of RES and inhibit cell proliferation in A2780 OC cells by regulating their target genes to participate in cell process-related signaling pathways. Our circRNA-miRNA-mRNA regulatory network has multiple ceRNA relationship pairs; this network reveals how the regulatory mechanism of RES influences the development of OC cells and provides a reference for selecting drug targets for OC treatment.

## Figures and Tables

**Table 1:** The primers of the reference genes.

Transcripts	Primers	
miR-212-x	F	AGACCTTGGCTCTAGACTGC
	RT	GTCGTATCCAGTGCGTGTCGTGGAGTCGGCAATTGCACTGGATACGACACAGTAA
hsa-miR-7-5p	F	CCCGTGGAAGACTAGTGATTT
	RT	GTCGTATCCAGTGCGTGTCGTGGAGTCGGCAATTGCACTGGATACGACAACAACA
novel-m0007-5p	F	GTGCGCTTGTTGTGATTCCT
	RT	GTCGTATCCAGTGCGTGTCGTGGAGTCGGCAATTGCACTGGATACGACAAAATGG
novel-m0002-3p	F	TGAGGCTGTGATGCTCTCCT
	RT	GTCGTATCCAGTGCGTGTCGTGGAGTCGGCAATTGCACTGGATACGACAGGGCTC
miR-541-X	F	CCCGAAAGGGATTCTGATGT
	RT	GTCGTATCCAGTGCGTGTCGTGGAGTCGGCAATTGCACTGGATACGACTGACCA
*U6*	F	CTCGCTTCGGCAGCACA
	RT	AACGCTTCACGAATTTGCGT

**Table 2:** Five differentially expressed miRNAs identified by sRNA-seq analysis.

sRNA	log_2_FoldChange	*p*-Value	FDR	Regulation
hsa-miR-7-5p	−1.48	2.98 × 10^−18^	2.96 × 10^−15^	Down
novel-m0002-3p	−1.74	3.90 × 10^−13^	1.11 × 10^−10^	Down
miR-541-x	−6.01	0.005796	0.06622	Down
miR-212-x	1.88	5.11 × 10^−13^	1.27 × 10^−10^	Up
novel-m0007-5p	−1.35	4.79 × 10^−10^	5.01× 10^−8^	Down

## Data Availability

The datasets presented in this study can be found in online repositories. The data availability statements can be found in NCBI BioProject PRJNA1119553.

## Supplementary Materials

The following supporting information can be downloaded at https://www.mdpi.com/article/10.3390/genes15070965/s1, Table S1: The total of miRNAs was identified in the two groups. Table S2: Differentially expressed miRNAs. Table S3: The total of circRNAs was identified in the two groups. Table S4: Differentially expressed circRNAs. Table S5: The total of mRNAs was identified in the two groups. Table S6: Differentially expressed mRNAs. Table S7: Table S4: The mRNAs and the corresponding circRNAs and miRNAs.

## References

[1] Viscarra T., Buchegger K., Jofre I., Riquelme I., Zanella L., Abanto M., Parker A.C., Piccolo S.R., Roa J.C., Ili C. (2019). Functional and transcriptomic characterization of carboplatin-resistant A2780 ovarian cancer cell line. Biol. Res..

[2] Shaik B., Zafar T., Balasubramanian K., Gupta S.P. (2021). An Overview of Ovarian Cancer: Molecular Processes Involved and Development of Target-based Chemotherapeutics. Curr. Top. Med. Chem..

[3] Stewart C., Ralyea C., Lockwood S. (2019). Ovarian cancer: An integrated review. Semin. Oncol. Nurs..

[4] Wang H., Liu L., Liu Q., Zheng J., Zheng Q., Chen Y., Xia H., Wu Q., Sun Y. (2023). Identification of upregulated exosomal miRNAs between A2780 and A2780/DDP human ovarian cancer cells by high-throughput sequencing. J. Ovarian Res..

[5] Yang L., Xie H.J., Li Y.Y., Wang X., Liu X.X., Mai J. (2022). Molecular mechanisms of platinum-based chemotherapy resistance in ovarian cancer (Review). Oncol. Rep..

[6] Tang M.Y., Ding D.N., Xie Y.Y., Shen F., Li J., Liu F.Y., Han F.J. (2023). Advances in mechanism of traditional Chinese medicine in inhibiting angiogenesis in ovarian cancer. Zhongguo Zhong Yao Za Zhi.

[7] Wang Y., Xie L., Liu F., Ding D., Wei W., Han F. (2024). Research progress on traditional Chinese medicine-induced apoptosis signaling pathways in ovarian cancer cells. J. Ethnopharmacol..

[8] Li H., Luo F., Jiang X., Zhang W., Xiang T., Pan Q., Cai L., Zhao J., Weng D., Li Y. (2022). CircITGB6 promotes ovarian cancer cisplatin resistance by resetting tumor-associated macrophage polarization toward the M2 phenotype. J. Immunother. Cancer.

[9] Rauf A., Imran M., Butt M.S., Nadeem M., Peters D.G., Mubarak M.S. (2018). Resveratrol as an anticancer agent: A review. Crit. Rev. Food Sci. Nutr..

[10] Vernousfaderani E.K., Akhtari N., Rezaei S., Rezaee Y., Shiranirad S., Mashhadi M., Hashemi A., Khankandi H.P., Behzad S. (2021). Resveratrol and colorectal cancer: A molecular approach to clinical researches. Curr. Top. Med. Chem..

[11] Almeida T.C., da Silva G.N., de Souza D.V., de Moraes Malinverni A.C., Aguiar O., Estadella D., Ribeiro D.A. (2021). Resveratrol effects in oral cancer cells: A comprehensive review. Med. Oncol..

[12] Hankittichai P., Thaklaewphan P., Wikan N., Ruttanapattanakul J., Potikanond S., Smith D.R., Nimlamool W. (2023). Resveratrol enhances cytotoxic effects of cisplatin by inducing cell cycle arrest and apoptosis in ovarian adenocarcinoma SKOV-3 cells through activating the p38 MAPK and suppressing AKT. Pharmaceuticals.

[13] Najafiyan B., Bokaii Hosseini Z., Esmaelian S., Firuzpour F., Rahimipour Anaraki S., Kalantari L., Hheidari A., Mesgari H., Nabi-Afjadi M. (2024). Unveiling the potential effects of resveratrol in lung cancer treatment: Mechanisms and nanoparticle-based drug delivery strategies. Biomed. Pharmacother..

[14] Fu X., Li M., Tang C., Huang Z., Najafi M. (2021). Targeting of cancer cell death mechanisms by resveratrol: A review. Apoptosis.

[15] Kleemann M., Schneider H., Unger K., Sander P., Schneider E.M., Fischer-Posovszky P., Handrick R., Otte K. (2018). MiR-744–5p inducing cell death by directly targeting HNRNPC and NFIX in ovarian cancer cells. Sci. Rep..

[16] Shi Z., Zhou X., Bao M., Jia R., Chu Y., Lin Y. (2022). miRNA-612 suppresses ovarian cancer cell tumorigenicity by downregulating NOB1. Am. J. Transl. Res..

[17] Feng S., Luo S., Ji C., Shi J. (2020). miR-29c-3p regulates proliferation and migration in ovarian cancer by targeting KIF4A. World J. Surg. Oncol..

[18] Yuan G., Ding W., Sun B., Zhu L., Gao Y., Chen L. (2021). Upregulated circRNA_102231 promotes gastric cancer progression and its clinical significance. Bioengineered.

[19] Chen R.X., Liu H.L., Yang L.L., Kang F.H., Xin L.P., Huang L.R., Guo Q.F., Wang Y.L. (2019). Circular RNA circRNA_0000285 promotes cervical cancer development by regulating FUS. Eur. Rev. Med. Pharmacol. Sci..

[20] Kristensen L.S., Jakobsen T., Hager H., Kjems J. (2022). The emerging roles of circRNAs in cancer and oncology. Nat. Rev. Clin. Oncol..

[21] Zhang M., Bai X., Zeng X., Liu J., Liu F., Zhang Z. (2021). circRNA-miRNA-mRNA in breast cancer. Clin. Chim. Acta.

[22] Zhao Y., Hu Y., Shen Q., Chen Q., Zhu X.J., Jiang S.S., Zhang Q. (2020). CircRNA_MYLK promotes malignant progression of ovarian cancer through regulating microRNA-652. Eur. Rev. Med. Pharmacol. Sci..

[23] Wei X., Lv H., Yang S., Yang X. (2021). CircRNA PLOD2 enhances ovarian cancer propagation by controlling miR-378. Saudi J. Biol. Sci..

[24] Pei C., Wang H., Shi C., Zhang C., Wang M. (2020). CircRNA hsa_circ_0013958 may contribute to the development of ovarian cancer by affecting epithelial-mesenchymal transition and apoptotic signaling pathways. J. Clin. Lab. Anal..

[25] Luo L., Gao Y.Q., Sun X.F. (2018). Circular RNA ITCH suppresses proliferation and promotes apoptosis in human epithelial ovarian cancer cells by sponging miR-10a-α. Eur. Rev. Med. Pharmacol. Sci..

[26] Ren B., Kwah M.X., Liu C., Ma Z., Shanmugam M.K., Ding L., Xiang X., Ho P.C., Wang L., Ong P.S. (2021). Resveratrol for cancer therapy: Challenges and future perspectives. Cancer Lett..

[27] Chen S., Zhou Y., Chen Y., Gu J. (2018). fastp: An ultra-fast all-in-one FASTQ preprocessor. Bioinformatics.

[28] Langmead B., Salzberg S.L. (2012). Fast gapped-read alignment with Bowtie 2. Nat. Methods.

[29] Kim D., Langmead B., Salzberg S.L. (2015). HISAT: A fast spliced aligner with low memory requirements. Nat. Methods.

[30] Memczak S., Jens M., Elefsinioti A., Torti F., Krueger J., Rybak A., Maier L., Mackowiak S.D., Gregersen L.H., Munschauer M. (2013). Circular RNAs are a large class of animal RNAs with regulatory potency. Nature.

[31] Pertea M., Pertea G.M., Antonescu C.M., Chang T.C., Mendell J.T., Salzberg S.L. (2015). StringTie enables improved reconstruction of a transcriptome from RNA-seq reads. Nat. Biotechnol..

[32] Robinson M.D., McCarthy D.J., Smyth G.K. (2010). edgeR: A Bioconductor package for differential expression analysis of digital gene expression data. Bioinformatics.

[33] Sun L., Luo H., Bu D., Zhao G., Yu K., Zhang C., Liu Y., Chen R., Zhao Y. (2013). Utilizing sequence intrinsic composition to classify protein-coding and long non-coding transcripts. Nucleic Acids Res..

[34] Li B., Dewey C.N. (2011). RSEM: Accurate transcript quantification from RNA-Seq data with or without a reference genome. BMC Bioinform..

[35] Ahmadi R., Ebrahimzadeh M.A. (2020). Resveratrol—A comprehensive review of recent advances in anticancer drug design and development. Eur. J. Med. Chem..

[36] He Y., Liu F., He M., Long F., Hu D., Chen J., Fang M., Wang Z. (2024). Molecular mechanism of resveratrol promoting differentiation of preosteoblastic MC3T3-E1 cells based on network pharmacology and experimental validation. BMC Complement. Med. Ther..

[37] Zhang Y., Yuan F., Li P., Gu J., Han J., Ni Z., Liu F. (2022). Resveratrol inhibits HeLa cell proliferation by regulating mitochondrial function. Ecotoxicol. Environ. Saf..

[38] Yao Y., Zhu J., Qin S., Zhou Z., Zeng Q., Long R., Mao Z., Dong X., Zhao R., Zhang R. (2022). Resveratrol induces autophagy impeding BAFF-stimulated B-cell proliferation and survival by inhibiting the Akt/mTOR pathway. Biochem. Pharmacol..

[39] Si L., Zhang M., Guan E., Han Q., Liu Y., Long X., Long F., Zhao R.C., Huang J., Liu Z. (2020). Resveratrol inhibits proliferation and promotes apoptosis of keloid fibroblasts by targeting HIF-1α. J. Plast. Surg. Hand Surg..

[40] Jang J., Song J., Lee J., Moon S.K., Moon B. (2021). Resveratrol Attenuates the Proliferation of Prostatic Stromal Cells in Benign Prostatic Hyperplasia by Regulating Cell Cycle Progression, Apoptosis, Signaling Pathways, BPH Markers, and NF-κB Activity. Int. J. Mol. Sci..

[41] Zhang Y., Yang S., Yang Y., Liu T. (2019). Resveratrol induces immunogenic cell death of human and murine ovarian carcinoma cells. Infect. Agents Cancer.

[42] Synowiec A., Brodaczewska K., Wcisło G., Majewska A., Borkowska A., Filipiak-Duliban A., Gawrylak A., Wilkus K., Piwocka K., Kominek A. (2023). Hypoxia, but Not Normoxia, Reduces Effects of Resveratrol on Cisplatin Treatment in A2780 Ovarian Cancer Cells: A Challenge for Resveratrol Use in Anticancer Adjuvant Cisplatin Therapy. Int. J. Mol. Sci..

[43] Liu Y., Tong L., Luo Y., Li X., Chen G., Wang Y. (2018). Resveratrol inhibits the proliferation and induces the apoptosis in ovarian cancer cells via inhibiting glycolysis and targeting AMPK/mTOR signaling pathway. J. Cell. Biochem..

[44] Dakal T.C., Kumar A., Maurya P.K. (2023). CircRNA-miRNA-mRNA interactome analysis in endometrial cancer. J. Biomol. Struct. Dyn..

[45] Sakshi S., Jayasuriya R., Ganesan K., Xu B., Ramkumar K.M. (2021). Role of circRNA-miRNA-mRNA interaction network in diabetes and its associated complications. Mol. Ther. Nucleic Acids.

[46] Ghazimoradi M.H., Babashah S. (2022). The role of CircRNA/miRNA/mRNA axis in breast cancer drug resistance. Front. Oncol..

[47] Yan J., Ye G., Jin Y., Miao M., Li Q., Zhou H. (2023). Identification of novel prognostic circRNA biomarkers in circRNA-miRNA-mRNA regulatory network in gastric cancer and immune infiltration analysis. BMC Genom..

[48] Liu X., Xiao H., Peng X., Chai Y., Wang S., Wen G. (2022). Identification and comprehensive analysis of circRNA-miRNA-mRNA regulatory networks in osteoarthritis. Front. Immunol..

[49] Sun S., Fang H. (2021). Curcumin inhibits ovarian cancer progression by regulating circ-PLEKHM3/miR-320a/SMG1 axis. J. Ovarian Res..

[50] Ma H., Qu S., Zhai Y., Yang X. (2022). Circ_0025033 promotes ovarian cancer development via regulating the hsa_miR-370-3p/SLC1A5 axis. Cell. Mol. Biol. Lett..

[51] Yu G., Li N., Zhao Y., Wang W., Feng X.L. (2018). Salidroside induces apoptosis in human ovarian cancer SKOV3 and A2780 cells through the p53 signaling pathway. Oncol. Lett..

[52] Huang J. (2021). Current developments of targeting the p53 signaling pathway for cancer treatment. Pharmacol. Ther..

[53] Zhao Y., Cai J., Shi K., Li H., Du J., Hu D., Liu Z., Wang W. (2021). Germacrone Induces Lung Cancer Cell Apoptosis and Cell Cycle Arrest via the Akt/MDM2/p53 Signaling Pathway. Mol. Med. Rep..

[54] Liu Y.L., Yang W.H., Chen B.Y., Nie J., Su Z.R., Zheng J.N., Gong S.T., Chen J.N., Jiang D., Li Y. (2021). miR-29b suppresses proliferation and induces apoptosis of hepatocellular carcinoma ascites H22 cells via regulating TGF-β1 and p53 signaling pathway. Int. J. Mol. Med..

[55] Tong X., Tang R., Xiao M., Xu J., Wang W., Zhang B., Liu J., Yu X., Shi S. (2022). Targeting cell death pathways for cancer therapy: Recent developments in necroptosis, pyroptosis, ferroptosis, and cuproptosis research. J. Hematol. Oncol..

[56] Chen L., Zhang X., Zhang Q., Zhang T., Xie J., Wei W., Wang Y., Yu H., Zhou H. (2022). A necroptosis related prognostic model of pancreatic cancer based on single cell sequencing analysis and transcriptome analysis. Front. Immunol..

[57] Long K., Gu L., Li L., Zhang Z., Li E., Zhang Y., He L., Pan F., Guo Z., Hu Z. (2021). Small-molecule inhibition of APE1 induces apoptosis, pyroptosis, and necroptosis in non-small cell lung cancer. Cell Death Dis..

[58] Mou Y., Wang J., Wu J., He D., Zhang C., Duan C., Li B. (2019). Ferroptosis, a new form of cell death: Opportunities and challenges in cancer. J. Hematol. Oncol..

[59] Wei L., Yin F., Zhang W., Li L. (2017). STROBE-compliant integrin through focal adhesion involve in cancer stem cell and multidrug resistance of ovarian cancer. Medicine.

[60] Yang L., Gu Y. (2023). SPTBN2 regulates endometroid ovarian cancer cell proliferation, invasion and migration via ITGB4-mediated focal adhesion and ECM receptor signalling pathway. Exp. Ther. Med..

[61] El-Kott A.F., Shati A.A., Ali Al-Kahtani M., Alharbi S.A. (2019). The apoptotic effect of resveratrol in ovarian cancer cells is associated with downregulation of galectin-3 and stimulating miR-424-3p transcription. J. Food Biochem..

[62] Ding J., Wen Y., Yuan X., He X. (2022). MicroRNA-mediated reprogramming of glucose, fatty acid and amino acid metabolism in cancer. Genome Instab. Dis..

[63] Wang Y., Yang H., Zhang G., Luo C., Zhang S., Luo R., Deng B. (2021). hsa-miR-7-5p suppresses proliferation, migration and promotes apoptosis in hepatocellular carcinoma cell lines by inhibiting SPC24 expression. Biochem. Biophys. Res. Commun..

[64] Zhang L., Zhang Y., Wang S., Tao L., Pang L., Fu R., Fu Y., Liang W., Li F., Jia W. (2020). MiR-212-3p suppresses high-grade serous ovarian cancer progression by directly targeting MAP3K3. Am. J. Transl. Res..

[65] Yue H., Liu L., Song Z. (2019). miR-212 regulated by HIF-1α promotes the progression of pancreatic cancer. Exp. Ther. Med..

[66] Zhou H., Zhang N. (2022). miR-212-5p inhibits nasopharyngeal carcinoma progression by targeting METTL3. Open Med..

[67] Zhao C., Li X., Pan X., Xu J., Jiang R., Li Y. (2023). LINC02532 by Mediating miR-541-3p/HMGA1 Axis Exerts a Tumor Promoter in Breast cancer. Mol. Biotechnol..

[68] Shao J.P., Su F., Zhang S.P., Chen H.K., Li Z.J., Xing G.Q., Liu H.J., Li Y.Y. (2020). miR-212 as potential biomarker suppresses the proliferation of gastric cancer via targeting SOX4. J. Clin. Lab. Anal..

[69] Wu X., Chen H., Zhang G., Wu J., Zhu W., Gu Y., He Y.I. (2019). MiR-212-3p inhibits cell proliferation and promotes apoptosis by targeting nuclear factor IA in bladder cancer. J. Biosci..

[70] Tang Y., Zhou L., Liu L. (2023). Circ_0085616 contributes to the radioresistance and progression of cervical cancer by targeting miR-541-3p/ARL2 signaling. Histol. Histopathol..

[71] Tang J., Wang R., Tang R., Gu P., Han J., Huang W. (2022). CircRTN4IP1 regulates the malignant progression of intrahepatic cholangiocarcinoma by sponging miR-541-5p to induce HIF1A production. Pathol. Res. Pract..

[72] Li D., Zhang J., Yang J., Wang J., Zhang R., Li J., Zhang R. (2021). CircMTO1 suppresses hepatocellular carcinoma progression via the miR-541-5p/ZIC1 axis by regulating Wnt/β-catenin signaling pathway and epithelial-to-mesenchymal transition. Cell Death Dis..

